# To What Extent Are the Effects of UV Radiation on Grapes Conserved in the Resulting Wines?

**DOI:** 10.3390/plants10081678

**Published:** 2021-08-15

**Authors:** María-Ángeles Del-Castillo-Alonso, Laura Monforte, Rafael Tomás-Las-Heras, Javier Martínez-Abaigar, Encarnación Núñez-Olivera

**Affiliations:** Faculty of Science and Technology, University of La Rioja, Madre de Dios 53, 26006 Logroño, La Rioja, Spain; maria-angeles-del.castillo@unirioja.es (M.-Á.D.-C.-A.); laura.monforte@unirioja.es (L.M.); rafael.tomas@unirioja.es (R.T.-L.-H.); encarnacion.nunez@unirioja.es (E.N.-O.)

**Keywords:** ultraviolet radiation, *Vitis vinifera* L. cv. Tempranillo, grapevine, wine, phenolic composition, volatile organic compounds

## Abstract

Ultraviolet (UV) radiation strongly influences grape composition, but only a few studies have focused on how this influence is conserved in the resulting wines. Here we analyzed to what extent the changes induced by exposing Tempranillo grapes to UV radiation from budbreak to harvest were conserved in wine. By using different cut-off filters and lamps, we differentiated the effects of ambient levels of UV-A and UV-B wavelengths, as well as the effects of a realistic UV-B enhancement associated with climate change. Among phenolic compounds, the most consistent responses to UV were those of flavonols (particularly quercetin-, kaempferol-, isorhamnetin- and myricetin-glycosides), which significantly increased in wines whose grapes had been exposed to a synergic combination of UV-A and UV-B radiation. This confirms that flavonols are the phenolic compounds most reliably conserved from UV-exposed grapes to wine, despite the possible influence of the winemaking process. Flavonols are important compounds because they contribute to wine co-pigmentation by stabilizing anthocyanins, and they are interesting antioxidants and nutraceuticals. Hydroxycinnamic acids also increased under the same UV combination or under UV-A alone. Wine VOCs were much less reactive to the UV received by grapes than phenolic compounds, and only esters showed significantly higher values under (mainly) UV-A alone. This was surprising because (1) UV-A has been considered to be less important than UV-B to induce metabolic changes in plants, and (2) esters are produced during winemaking. Esters are relevant due to their contribution to the fruity aroma in wines. In general, the remaining phenolic compounds (stilbenes, flavanols, hydroxybenzoic acids, and anthocyanins) and VOCs (alcohols, hydrocarbons, and fatty acids), together with wine color and antioxidant capacity, showed inconsistent or non-significant responses to UV radiation. These results were summarized by a multivariate analysis. Our study opens up new possibilities to artificially manipulate UV radiation in grapevine cultivation to improve both grape and wine quality.

## 1. Introduction

The solar spectrum includes three wavelength bands of ultraviolet (UV) radiation: UV-C (100–280 nm), UV-B (280–315 nm) and UV-A (315–400 nm). Only wavelengths greater than 290 nm reach the biosphere, with UV-A representing the major fraction (95%) whereas UV-B constitutes the remaining 5%. At ground level, UV radiation represents around 5% of photosynthetically active radiation (PAR, 400–700 nm) [1]. UV radiation (particularly UV-B) has traditionally been considered as a general stressor for plants, and its excess leads to diverse alterations in DNA, hormones, and photosynthesis (pigment degradation, photoinhibition, and reductions in quantum yield, net photosynthesis, and Calvin cycle enzyme activities), many of them caused by oxidative damage [2,3]. However, real ambient UV levels cause regulation and acclimation processes [1,4]. On the other hand, UV manipulation represents an innovative technological tool to improve crop quality through modifying, among other characteristics, the plant architecture, metabolite contents, and pest and disease tolerance [5].

Grapevine (*Vitis vinifera* L.) has been widely investigated regarding UV effects [6,7]. In grapevine leaves, short high-dose UV exposures can lead to diverse damage, whereas long exposures at either dose hardly affected most photosynthetic and biochemical parameters, probably due to acclimation processes such as the increase in UV-absorbing compounds and antioxidant capacity [6]. In grapes, UV radiation causes the accumulation of several phenolic compounds (see below) and the induction of related genes [6,8], while UV effects on agronomic variables, such as berry size and yield, are far from clear [9].

UV effects on grapes are commercially important, and knowledge of this subject is increasing rapidly. Different studies on grapes have mainly applied four experimental approaches: (1) using UV natural gradients along latitude [10] or altitude [11]; (2) using cut-off filters to exclude ambient UV radiation, thus assessing the effects of ambient UV levels [12,13,14,15]; (3) using lamps to increase UV-A and/or UV-B exposures, trying to simulate stratospheric ozone degradation or to improve grape characteristics, particularly the contents of metabolites determining grape and wine quality [9,15,16], and (4) using UV-C to increase specific metabolites, particularly stilbenes, under controlled conditions [17,18,19]. The most studied metabolites in these regards have been phenolic compounds and, to a lesser extent, volatile organic compounds (VOCs). Phenolic compounds are chemically diverse, from relatively simple phenolic acids (hydroxycinnamic and hydroxybenzoic acids, and their derivatives) to stilbenes and different types of flavonoids (flavanols, flavonols, and anthocyanins) [20]. Many of them have nutraceutical properties. Regarding VOCs, they are equally diverse, including (among others) alcohols, hydrocarbons, esters, aldehydes, terpenes, and fatty acids [20,21]. All these compounds contribute to the different colors, aromas, flavors, and textures of grapes and, especially, wines.

Despite the notable background of knowledge already available to the scientific community, only a few studies have evaluated to what extent the effects of UV radiation on grapes are conserved in the resulting wines [11,16,22,23]. These studies were carried out on three different varieties (Malbec, Pinot noir, and Tempranillo), either using lamps to expose grapes to enhanced UV-B levels [16], or covering grapes with UV or UV-B filters to study the effects of ambient levels [11,22,23]. Response variables measured in wines were diverse: only overall variables (such as color, total phenolics, and total anthocyanins) [11], individual VOCs [22], individual phenolic compounds [23], or both types of compounds [16]. Hence, no study to our knowledge has tested how the exposure of grapes to different UV wavelengths (specifically, UV-A, UV-B, or a combination of both) may determine the characteristics of the resulting wines.

In the context described, our aim was to study to what extent the effects of UV radiation on grapes were conserved in the resulting wines, differentiating the effects of ambient UV-A and UV-B levels, as well as the effects of the enhanced UV-B levels that will probably reach the biosphere as a consequence of ozone degradation and climate change [24]. Regarding this approach, the application of enhanced UV-B over ambient levels could also serve to test the possibilities of using UV as a technological tool in viticulture and enology. To assess the UV effects, we measured variables directly related to the quality of grapes and wines, including individual phenolic compounds and VOCs.

## 2. Results

Grapevine plants were exposed to five different radiation regimes from budbreak to harvest (see Section 4.1): P (only photosynthetically active radiation, PAR), PA (PAR + UV-A), PB (PAR + UV-B), PAB (PAR + UV-A + UV-B), and PAB↑ (PAR + UV-A + enhanced UV-B). At the end of the exposure period, phenolic compounds and VOCs were measured in both grapes [9] and the resulting wines (Table 1 and Table 2). A comparison of the results obtained in grapes and wines is shown in Table 3.

### 2.1. Main Effects of UV Radiation on Grapes

At harvest, UV-B levels caused stronger effects on grapes than ambient UV-A, although some synergic effects between UV-B and UV-A were observed [9]. These effects included increases in flavonol contents, particularly quercetins and kaempferols, whereas the responses of anthocyanins, stilbenes, flavanols, phenolic acids, and VOCs were more diffuse or nonexistent (Table 3). Regarding VOCs, one hydrocarbon (heptane,2,2,4,6,6-pentamethyl) increased in PAB↑ samples, while four fatty acids (2-ethylhexanoic, heptanoic, octanoic, and nonanoic acids) increased in both PB and PAB↑ samples. On the other hand, enhanced UV-B (PAB↑ samples) led to rather subtle changes in comparison with ambient UV-B (PAB samples), but differences between the radiation regimes could be demonstrated by a multivariate analysis.

### 2.2. Effects of the Radiation Received by Grapes on the Phenolic Composition of the Resulting Wines

**Figure 1:**
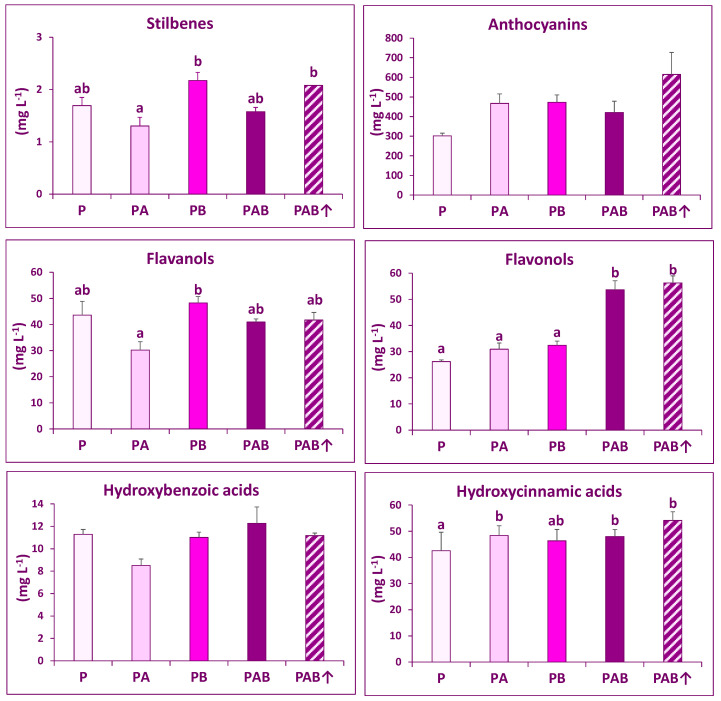
Contents of phenolic families in the wines made from grapes exposed to five different radiation regimes: P (only PAR), PA (PAR + UV-A), PB (PAR + UV-B), PAB (PAR + UV-A + UV-B), and PAB↑ (PAR + UV-A + enhanced UV-B). For each variable, different letters indicate significant differences between radiation regimes (post hoc Tukey test after a one-way ANOVA test using radiation regime as main factor). Means ± SE are shown.

A total of 45 phenolic compounds were identified in wines (Table 1), 33 of which were also found in grapes (Table 3). The most abundant phenolic group was anthocyanins, followed by flavanols, flavonols, and hydroxycinnamic acids (in similar amounts), and finally hydroxybenzoic acids and stilbenes (Figure 1). The total concentrations of stilbenes, flavanols, flavonols, and hydroxycinnamic acids were significantly affected by the radiation regime. The effect of UV radiation on total stilbenes and total flavanols was rather inconsistent, because the only significant differences found were those established between PB samples (and PAB↑ samples for stilbenes) and PA samples, whereas P samples, which received insignificant UV-B amounts [9], were not different from samples exposed to some UV-B (PB, PAB, and PAB↑). The response of total hydroxycinnamic acids was more consistent, given that samples of three radiation regimes with UV-A or a combination of UV-A and UV-B (PA, PAB, and PAB↑), showed higher concentrations than P samples, although differences were relatively low. The most consistent responses were those of total flavonols, which significantly increased in PAB and PAB↑ samples in comparison with the remaining samples. The total anthocyanins and hydroxybenzoic acids showed no significant responses to radiation regimes.

Regarding individual compounds (Table 1), 18 of them (40% of the total) were significantly influenced by the radiation regime (10 flavonols, four anthocyanins, three flavanols, and one hydroxycinnamic acid). As described for the total concentrations of phenolic groups, about half of these responses (those of flavanols, the hydroxycinnamic acid, and most anthocyanins) did not reflect a consistent effect of UV radiation on wines, because (1) significant differences were found only between pairs of samples (P and PA, P and PB, PB and PAB, or PA and PAB↑); or (2) P samples showed similar values to samples that had received significant UV amounts. In contrast, the responses of most flavonols and the anthocyanin delphinidin-glucoside to UV were more consistent. Specifically, the concentrations of three minor (kaempferol-glucoside, kaempferol-glucuronide, and isorhamnetin-glucuronide) and four major (three quercetin-glycosides and myricetin-glucuronide) flavonols were higher in PAB and PAB↑ samples than in other regimes (at least P, but also PA and PB in most cases).

No significant difference between PAB and PAB↑ samples was found for the total concentrations of phenolic families or for individual phenolic compounds (Figure 1, Table 1).

Some comprehensive phenolic variables, such as total phenols and the bulk levels of UVACs (Table 1), showed no significant response to the radiation regime. The total polyphenol index showed significant differences only between P and PA samples, with higher values in the former than in the latter samples, whereas the remaining samples showed intermediate values.

### 2.3. Effects of the Radiation Received by Grapes on the VOCs of the Resulting Wines

**Figure 2:**
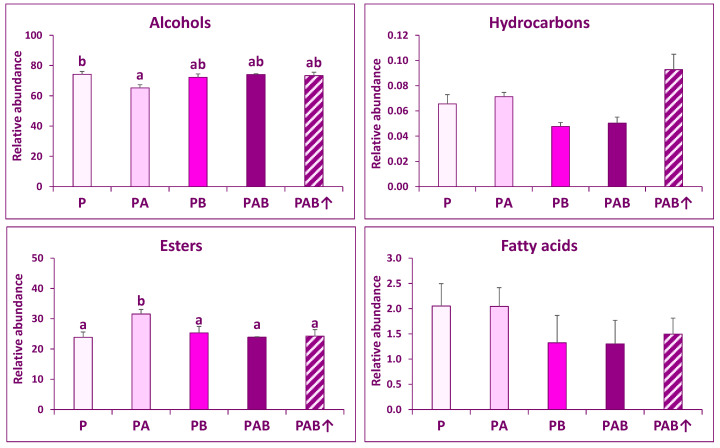
Relative abundance (percentages) of families of volatile organic compounds (VOCs) in wines made from grapes exposed to five different radiation regimes: P (only PAR), PA (PAR + UV-A), PB (PAR + UV-B), PAB (PAR + UV-A + UV-B), and PAB↑ (PAR + UV-A + enhanced UV-B). For each variable, different letters indicate significant differences between radiation regimes (post hoc Tukey test after a one-way ANOVA test using radiation regime as main factor). Means ± SE are shown.

A total of 30 VOCs were identified: 18 esters, four alcohols, four fatty acids, two hydrocarbons, one lactone, and one aldehyde. Only six of these individual VOCs were also found in grapes (Table 3). The most abundant VOCs were alcohols (approximately 65−75% of total VOCs), followed by esters (25−35%), fatty acids (around 2%), and hydrocarbons (less than 0.1%). The total concentrations of alcohols and esters were significantly affected by the radiation regime (Figure 2). Regarding total alcohol concentrations, PA samples showed significantly lower values than P samples, while the remaining samples were not significantly different from either P or PA samples. As for total ester concentrations, PA samples showed significantly higher values than the remaining samples, which showed similar values. Total hydrocarbons and fatty acids showed no significant response to radiation regimes.

Fifteen individual VOCs (50% of the total) were significantly influenced by the radiation regime: eight esters, three fatty acids, two alcohols, one hydrocarbon, and one aldehyde (Table 2). Wine samples made from grapes that had received some kind of UV radiation showed higher concentrations of some specific VOCs, such as ethyl decanoate, ethyl octanoate, methyl octanoate, hexanoic acid, octanoic acid, nonanoic acid, 2-methyl-1-butanol, and tetradecane. This suggested some effect of either UV-A, UV-B, or the combination of both. However, for other VOCs (ethyl dodecanoate, ethyl nonanoate, tetradecanoic acid ethyl ester, 3-methyl butanol, and n-nonaldehyde), concentrations in P samples were higher than (or similar to) those found in samples made with UV-exposed grapes.

No significant difference between PAB and PAB↑ samples was found for the total concentrations of VOCs families (Figure 2), but a few individual VOCs did show differences (Table 2): 2-methyl-1-butanol and nonanoic acid concentrations were higher in PAB↑ than in PAB samples, whereas 3-methyl butanol, ethyl nonanoate, and n-nonaldehyde showed opposite results.

### 2.4. Effects of the Radiation Received by Grapes on Other Wine Variables

The effect of the radiation received by grapes on the color of the resulting wines was not significant. The effect on the antioxidant capacity was, although significant, rather inconsistent, because the antioxidant capacity was lower in PA samples than in P, PB, and PAB samples, while PAB↑ samples showed no difference to any other type of sample.

### 2.5. Summarizing the Effects of UV Radiation on Wines by PCA 

**Figure 3:**
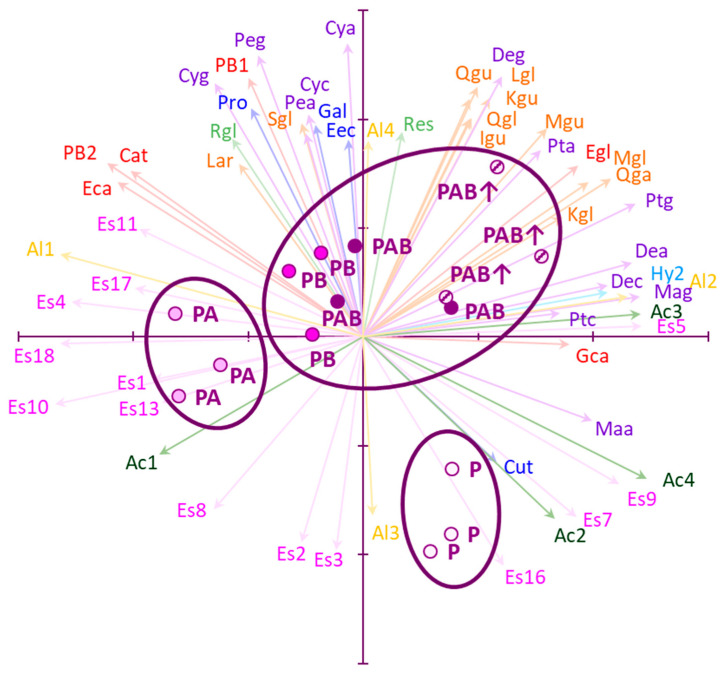
Ordination, through principal components analysis (PCA), of wines made from grapes exposed to five different radiation regimes: P (only PAR), PA (PAR + UV-A), PB (PAR + UV-B), PAB (PAR + UV-A + UV-B), and PAB↑ (PAR + UV-A + enhanced UV-B). PCA was based on the content of individual phenolic compounds and volatile organic compounds (VOCs). Biological replicates of treatments were used for ordination. Significant loading factors are shown as arrows. Ac1, ethanoic acid; Ac2, hexanoic acid; Ac3, nonanoic acid; Ac4, octanoic acid; Al1, 3-methyl butanol; Al2, 2-methyl-1-butanol; Al3, 1-hexanol; Al4, phenyl ethyl alcohol; Cat, catechin; Cut, coumaroyl tartaric acid; Cya, cyanidin-(6′-acetyl)glucoside; Cyc, cyanidin-(6′-p-coumaroyl)glucoside; Cyg, cyanidin-glucoside; Dea, delphinidin-(6′-acetyl)glucoside; Dec, delphinidin-(6′-p-coumaroyl)glucoside; Deg, delphinidin-glucoside; Eca, epicatechin; Eec, caffeic acid ethyl ester; Egl, epigallocatechin; Es1, ethyl butanoate; Es2, methyl hexanoate; Es3, ethyl hexanoate; Es4, ethyl heptanoate; Es5, methyl octanoate; Es7, ethyl octanoate; Es8, ethyl nonanoate; Es9, ethyl decanoate; Es10, ethyl dodecanoate; Es11, ethyl hexylsalicylate; Es13, 2-methylbutyl acetate; Es16, octanoic acid, 3-methylbutyl ester; Es17, hexadecanoic acid, ethyl ester; Es18, tetradecanoic acid ethyl ester; Gal, gallic acid; Gca, gallocatechin; Hy2, tetradecane; Igu, isorhamnetin-glucuronide; Kgl, kaempferol-glucoside; Kgu, kaempferol-glucuronide; Lar, laricitrin; Lgl, laricitrin-glucoside; Maa, malvidin-(6′-acetyl)glucoside; Mag, malvidin-glucoside; Mgl, myricetin-glucoside; Mgu, myricetin-glucuronide; PB1, procyanidin B1; PB2, procyanidin B2; Pea, peonidin-(6′-acetyl)glucoside; Peg, peonidin-glucoside; Pro, protocatechuic acid; Pta, petunidin-(6′-acetyl)glucoside; Ptc, petunidin-(6′-p-coumaroyl)glucoside; Ptg, petunidin-glucoside; Qga, quercetin-galactoside; Qgl, quercetin-glucoside; Qgu, quercetin-glucuronide; Res, resveratrol; Rgl, resveratrol-glucoside; Sgl, syringetin-glucoside. The different families of compounds are shown in different colors: stilbenes in light green, flavanols in red, flavonols in orange, phenolic acids in deep blue, anthocyanins in violet, alcohols in yellow, hydrocarbons in light blue, esters in pink, and fatty acids in deep green. Axis I is the horizontal one, and axis II is the vertical one. Each tick on the axes represents one unit.

In the PCA performed using the phenolic compounds and VOCs of wines (Figure 3), the accumulated variance by the first two axes was 51% (28% for axis I, and 23% for axis II). Loading factors for the positive part of axis I were mainly flavonols and anthocyanins, together with one fatty acid (nonanoic acid) and one alcohol (2-methyl-1-butanol), and for the negative part, several esters. Loading factors for the positive part of axis II were flavonols, anthocyanins, phenolic acids, and stilbenes, and for the negative part, some esters (different from those acting as loading factors for axis I) and fatty acids. Loading factors were evidently concentrated towards the positive parts of both axes, as can be seen from the density of arrows. Wine samples were clearly ordinated according to the radiation regime to which grapes had been exposed, and replicates of each regime clustered more or less together, confirming the consistency of the results. P samples, elaborated with non-UV-exposed grapes, were placed in the fourth quadrant, clearly separated from the remaining samples. This separation was due to the relatively low concentrations of flavonols and anthocyanins, and the relatively high concentrations of fatty acids, in P samples. Among the wines made from UV-exposed grapes, PA samples were clustered on the more negative part of axis II because of their high concentrations of several esters, and their relatively low concentrations of flavonols. PB, PAB, and PAB↑ samples, all of which had been made with grapes exposed to UV-B or a combination of UV-B and UV-A, were distributed along the positive (and less negative) part of axis I, in the first and second quadrants of the plot. PB samples were closer to PA samples than PAB and PAB↑ samples. Clearly, the higher concentrations of flavonols, anthocyanins, and hydrocarbons, displaced both PAB and PAB↑ samples towards the positive part of axes I and II. PAB and PAB↑ replicates were not totally separated by PCA, but remained somewhat mixed.

### 2.6. Comparing the Effects of UV Radiation on Grapes and Wines

A total of 51 variables related to phenolic composition and VOCs could be compared between grapes [9] and the resulting wines (Table 3). Only five of them showed a similar effect of the radiation regimes used on both grapes and wines: total flavonols, four individual flavonols (kaempferol-glucoside and three quercetin-glycosides), and one fatty acid (nonanoic acid). In all these cases, samples exposed to a combination of UV-A and UV-B radiation (either PAB or PAB↑ samples, or both), showed higher concentrations of those compounds than samples exposed to only PAR, or to a combination of PAR and either UV-A or UV-B (P, PA, and PB samples). In the remaining 46 variables, grapes and the respective resulting wines showed different responses (or no response) to the radiation regimes.

## 3. Discussion

The present study is one of few evaluating how the UV radiation received by grapes determines differences in the resulting wines, taking into consideration diverse variables, such as phenolic compounds, VOCs, antioxidant capacity, and sensorial characteristics (color). In addition, this is the first study to our knowledge differentiating between the effects of UV-A and UV-B wavelengths from grapes to wine.

### 3.1. Wine Phenolic Composition

Wine total flavonols, together with four major (three quercetin- and one myricetin-glycosides) and three minor (two kaempferol- and one isorhamnetin-glycosides) individual flavonols, showed the most consistent responses to the UV radiation applied to the grapes. Hence, flavonols were the phenolic compounds more reliably conserved from grapes to wine. In general, these variables significantly increased in PAB and PAB↑ samples in comparison with the remaining samples, including those whose grapes had received either UV-A or UV-B alone (PA and PB samples, respectively). These consistent responses could be expected, because the accumulation of different flavonols, especially glycosylated quercetins, is the most reliable response of grapes [6,8,10,15,23,25,26,27,28,29,30] and the resulting wines [16,23] to UV radiation, across different grapevine varieties and experimental conditions. Given that flavonols contribute to wine co-pigmentation by stabilizing anthocyanins [31], and they are also interesting antioxidants and nutraceuticals [32,33], their increase under UV radiation can improve the quality of both grapes and wines. It is interesting to highlight that the responses of flavonols were determined by a synergic effect between UV-A and UV-B, as found in other studies dealing only with grapes [9,26,34]. Consequently, this is the first time to our knowledge that this synergy has extended from grapes to the resulting wines, thus proving the effect of UV radiation on grapevines.

The response of wine total hydroxycinnamic acids to UV was significant, and PA, PAB, and PAB↑ samples showed higher concentrations than P samples. Nevertheless, differences between regimes were relatively low and the response of total hydroxycinnamic acids was not clearly reflected in any individual acid. The response of total hydroxycinnamic acids, although subtle, was surprising because these compounds do not usually respond to UV radiation, neither in grapes [8,11,14,23,35,36] nor in the resulting wines [23]. This lack of response is likely due to the competition between flavonoids and phenolic acids for the same precursors in their synthesis. In addition, some hydroxycinnamic acids are produced de novo during winemaking, which can decouple, to a certain extent, the relationship between their contents in grapes and their concentrations in wines. However, high UV-B irradiance increased the total hydroxycinnamic acids in both Tempranillo grapes and the resulting wines [16], showing that UV-B levels well over the ambient ones may be needed to induce the accumulation of these phenolic compounds. Given the importance of hydroxycinnamic acids and their derivatives to the color, taste, and flavor of wines [21], further research is needed to better understand their responses to UV radiation. 

Total anthocyanins did not respond to radiation regimes and, among the 15 individual anthocyanins measured, only delphinidin-glucoside showed a consistent response to UV, with higher concentrations in PAB↑ than in P and PA wines. Overall, the diffuse response of anthocyanins to UV radiation was expected, being consistent with most previous results obtained in grapes and, occasionally, in wines [16]. Nevertheless, anthocyanins increased in wines made with Pinot noir and Tempranillo grapes exposed to ambient UV [22,23]. Thus, the responses of grape anthocyanins to UV are complex and may depend on the interaction of internal (variety, berry development) and environmental (temperature, radiation) factors [6]. In addition, anthocyanins can change during winemaking due to polymerization and/or degradation [37], which may limit the relationship between grape and wine anthocyanins. Consequently, although they are crucial compounds for the red coloring of wine, and also have nutraceutical properties, the effects of UV radiation on grape and, particularly, wine anthocyanins are not fully understood yet.

The effect of UV radiation on wine total stilbenes was inconsistent and not supported by any individual stilbene. Stilbenes respond to both biotic and abiotic stressors (including pathogen attacks and UV-C radiation) in grapes [17,18,19], and they also vary along berry development and during vinification [7,21]. However, their responses to other UV wavelengths than UV-C in grapes and the resulting wines ranged from significant [9,11,23] to negligible [8,14,16]. As occurs with other compounds, this topic requires more research, because stilbenes, and specifically resveratrol, are considered health-promoting compounds [21].

The responses of total and individual flavanols in wine to ambient UV levels were incongruent or non-significant, which agrees with previous results obtained in both grapes and wine [23]. However, high UV-B levels increased several individual flavonols in grapes and/or wines [16]. Thus, as occurred with hydroxycinnamic acids, flavanol induction in grapes and further persistence in wines may require higher-than-ambient UV levels. This is important because flavanols contribute to wine’s astringency and bitterness [31] and have antioxidant and anticarcinogenic properties. Hydroxybenzoic acids showed no significant response to radiation regimes, which agrees with previous results [23].

Total polyphenol index, total phenols, and the bulk levels of UVACs showed no definitive response to UV radiation. This may reflect, to a certain extent, the lack of response of most phenolic compounds, including the most abundant ones (anthocyanins), and also the fact that these comprehensive variables integrate the individual responses of many different compounds, which can be diverse and even contradictory. Nevertheless, in some studies, total polyphenol index and total phenols increased (significantly or not) in wines made with grapes previously exposed to ambient UV levels [22,23], but this increase did not occur in other studies [11,16]. Factors underlying this variability could be the grapevine variety, UV levels applied, and winemaking process.

### 3.2. Wine VOCs

The radiation regime significantly affected 15 out of 30 wine individual VOCs (50%), among which eight (27%) increased in wines made from grapes exposed to UV-A, UV-B, or both. In other studies, the proportions of compounds affected by UV radiation were similar: seven out of 27 (26%), among which six compounds (22%) increased under high UV-B [16], and 13 out of 50 (26%), although only three (6%) increased under ambient UV [22]. Given the importance of VOCs for wine aroma, these overall figures are promising for the potential manipulation of UV as a technological tool in grapevine cultivation and winemaking.

Total esters showed a specific effect of UV-A alone, and their concentrations increased in PA in comparison with the remaining samples, whose values were similar. Although most esters are by-products of yeast metabolism during fermentation, they can be modified by the grape composition [22], which in turn can be influenced by UV radiation. This would explain the significant influence of UV-A on wine esters in our study. Additionally, the presence of UV-B (PB, PAB, and PAB↑ samples) seemed to have some overall inhibitory effect on total ester accumulation. Thus, UV-B would counteract the promoting effect of UV-A. Nevertheless, one major (ethyl octanoate) and two minor (ethyl decanoate and methyl octanoate) individual esters showed higher concentrations in wine samples made from grapes exposed to both UV-A and UV-B, in comparison with wines made from non-exposed grapes, which brings into question the inhibitory role of UV-B. The effects of the UV received by grapes on the esters of the resulting wines are controversial. The concentrations of seven esters in wines made from Pinot noir grapes exposed to ambient UV (both UV-A and UV-B), were lower than those made from grapes exposed to UV-deprived solar radiation [22]. This would agree with a potential inhibitory role of UV-B against ester accumulation. However, the total esters did not change in wines made from Tempranillo grapes exposed to high UV-B, and three individual esters even increased [16]. The influence of variety, UV treatment, and vinification process may explain these differences, but further research is needed to better understand the effect of UV-A and UV-B on wine esters. Esters are important because they contribute to fruity aroma in wines; specifically, ethyl octanoate has been considered an indicator of red wine quality [21].

Wine samples made from grapes that had received some UV (mainly PA and PAB↑ samples) showed higher concentrations of three fatty acids (hexanoic, octanoic, and nonanoic acids). Hexanoic acid also increased in wines made from grapes exposed to high UV-B [16], but octanoic acid decreased in wines made from grapes exposed to ambient UV [22]. Thus, the influence of UV on wine straight-chain fatty acids is far from clear. These compounds are produced during wine fermentation and their excessive presence in wine is associated with fatty, cheesy, and rancid aromas, although low concentrations contribute to aroma complexity [22]. Hence, a potential increase in these compounds under UV should be carefully monitored. 

The major alcohol 2-methyl-1-butanol significantly increased in PAB↑ samples, but was not influenced by UV radiation in other studies [16,22]. Given the importance of alcohol concentrations in wines, the elevation of 2-methyl-1-butanol under supplemental UV-B warrants more study. The last VOC increasing in wines under UV radiation was the hydrocarbon tetradecane, showing higher concentrations in PAB↑ than P and PB samples. No comparative results are available for this compound, but, being a minor component of our wines, its increase would potentially have a low impact on wine quality.

Other comprehensive VOC variables (total alcohols, hydrocarbons, and fatty acids), as well as many individual VOCs, showed unclear or non-significant responses to UV radiation. Based on the few comparative studies available [16,22], the responses of wine VOCs to the UV received by grapes do not follow any common pattern, and more research is needed to understand these processes. In our study, VOCs showed a more modest response to UV than phenolic compounds, and thus their influence in the ordination of the samples by PCA was lower (Figure 3). 

### 3.3. Other Wine Variables

In our study, the effect of the UV received by grapes on the color of the resulting wines was not significant, probably reflecting the weak response of anthocyanins [16]. Previous results obtained on this subject are diverse. Ambient UV increased both anthocyanins and color in Pinot noir wines [22], and anthocyanins but not color in Tempranillo wines [23], while high UV-B did not change either anthocyanins or color in Tempranillo [16] and Malbec [11] wines. The effect of UV on the wine antioxidant capacity was inconsistent, despite the increase in several dihydroxylated (quercetins) and trihydroxylated (myricetins) flavonols, whose antioxidant capacity is higher than that of monohydroxylated kaempferols or isorhamnetins. This lack of clear response is contrasted with the increase in antioxidant capacity of wines made from grapes exposed to ambient UV and high UV-B [16,23]. Given the commercial importance of wine color and the health implications of wine antioxidant activity, these discrepancies should be better studied.

### 3.4. Summarizing the Effects of UV Radiation on Wines 

The results described above were summarized through a PCA (Figure 3). PCA corroborated the strong influence of wine flavonols on the ordination of samples, as expected on the basis of previous studies [16]. Flavonols were loading factors for the positive part of axes I and II, which led to the clear grouping of PAB↑ samples in the first quadrant. Thus, the combination of UV-A and enhanced levels of UV-B was the UV treatment most associated with flavonol concentrations, whereas the remaining treatments were less or not associated with these variables. On the other hand, the influence of wine esters on the clear separation of PA samples in the PCA plot was surprising, because these compounds are produced during vinification and their responses to the UV radiation received by grapes were unclear in previous studies [16,22]. Overall, the relationship of UV-A radiation with two groups of compounds determining wine quality (flavonols and esters) is curious because UV-A has frequently been ignored in grapevine experimentation, despite its effects on other plants [38]. Hence, it would be recommendable to consider UV-A in future studies on grapevines. Other diverse phenolic (anthocyanins, flavanols, phenolic acids, and stilbenes) and volatile (fatty acids, 2-methyl-1-butanol) compounds were significant loading factors in the PCA performed, but their effects on the ordination of samples were lower than those of flavonols and esters. 

Another relevant point regarding PCA ordination is that P samples were placed in the fourth quadrant, clearly separated from the remaining samples. This provides further evidence of the crucial influence of the UV radiation received by grapes on the composition of wines. Nevertheless, the specific influence of the different UV wavelengths and levels used in our study was complex, because one PAB replicate was intermixed with PAB↑ samples, whereas the remaining two PAB replicates and all the PB samples were located in an intermediate place between PAB↑ and PA samples. Diverse loading factors were responsible for this ordination, and more experimentation is needed to disentangle the specific role of both UV wavelengths and levels on wine composition and quality.

### 3.5. Effects of a Realistic UV-B Enhancement on Wine Composition

Differences between PAB and PAB↑ samples were scarce, and both types of samples were somewhat mixed in the PCA plot (Figure 3). Thus, a realistic 10% UV-B enhancement [24] did not change the grape composition [9] as strongly as needed to be reflected in the resulting wines. This could be due to the Mediterranean origin of the cultivar used [39] and its concomitant adaptation to high UV-B levels, which would lead to no additional effect of a UV-B supplement on the composition of grapes and the resulting wines. Probably, higher short-term peak irradiances instead of modestly higher UV-B doses would have caused stronger changes in both grapes and wines [16]. More experiments using different doses and irradiance levels are needed to confirm or reject this hypothesis.

### 3.6. Comparing the Effects of UV on Grapes and the Resulting Wines

The effects of UV on both grapes and the resulting wines were compared by measuring a total of 51 variables related to phenolic compounds and VOCs (Table 3). Among the 51 variables, only five showed similar significant effects on grapes and wines. This low number of coincidences may not be strange because, at first sight, the transformations occurring during winemaking would weaken the relationship between grape and wine compounds. Nevertheless, some of the most conspicuous UV-induced changes in grapes were indeed reflected in the resulting wines, which showed increased concentrations of total flavonols, kaempferol-glucoside, and three quercetin-glycosides in PAB and/or PAB↑ samples in comparison with the remaining samples (Table 3). In addition, a fatty acid (nonanoic acid) also showed similar responses in grapes and wines. It is difficult to compare these results with previous findings because of the scarcity of studies considering the effects of UV radiation on both grapes and the resulting wines. Moreover, in two of the existing studies, the variables measured in grapes were not measured in wines and vice versa [11,22], and thus no comparison between grapes and wines is possible. More interesting results can be derived from the two remaining studies, carried out on Tempranillo variety, because (1) ambient UV increased 12 phenolic variables in both grapes and wines, including the five UV-reactive variables that also increased in our study [23], and (2) high UV-B increased total flavonols in both grapes and wines [16]. These results confirm that the increase in flavonols is the most reliable response of both grapes and the resulting wines to different UV conditions, including a sufficient dose of a combination of UV-A and UV-B radiation [23], or an irradiance peak of only UV-B [16]. 

## 4. Materials and Methods

### 4.1. Plant Material, Culture Conditions, and Experimental Design

This experiment was conducted in 2017 in an experimental vineyard located at the University of La Rioja (Logroño, La Rioja, northern Spain, 42°27′ N, 2°25′ W, 373 m elevation). The experiment was performed on *Vitis vinifera* L. cv. Tempranillo (clone 43) plants grafted onto 110R rootstock and planted in 50-L pots in 2013. Tempranillo is the third most used cultivar worldwide and is expanding rapidly [40].

Plants were exposed to five different radiation regimes:

- P (photosynthetically active radiation, PAR, alone), using XT Vitroflex 395 Solarium Incoloro (Polimertecnic, Girona, Spain), which blocked all UV radiation.
- PA (PAR + UV-A), using acetate Folex 320 (Folex GmbH, Dreieich, Germany) complemented with a polymetacrylate rigid filter (PMMA XT Vitroflex 295, Polimertecnic, Girona, Spain). These filters blocked UV-B and UV-C radiation.
- PB (PAR + UV-B), using a Vitroflex 395 filter and UV-B lamps (TL 40W/12 UVB, Philips Lighting, Madrid, Spain). Lamps were switched on for 10 min periods in the middle hours of the day to provide the plants with the same UV-B that they would receive if exposed to ambient sunlight.
- PAB (PAR + UV-A + UV-B), using PMMA XT Vitroflex 295 (Polimertecnic, Girona, Spain), which blocked UV-C radiation.
- PAB↑ (PAR + UV-A + enhanced UV-B), using the same filter as in PAB and the same lamps as in PB, but providing 10% higher UV-B than that received in the PAB treatment by adjusting the time of functioning of the lamps. This UV-B enhancement was realistic and compatible with the predictions of global climate change [24].

Three replicates were established for each regime, each consisting of two plants. The experiments took place from 6 April (before bud break) to 5 September (harvest). Culture conditions and details on the UV irradiances and doses applied in each regime were as described in a previous study [9].

### 4.2. Grape Sampling, Winemaking, and Wine Analysis 

For each radiation regime and replicate, grapes were collected at harvest (5 September) and analyzed for phenolic compounds and volatile organic compounds (VOCs), as described in a previous study [9]. Additional grapes were separately collected for winemaking. Grapes were destemmed and crushed, and alcoholic fermentation was performed [23]. Around 3 kg of crushed grapes were introduced into 2.5 L glass bottles. Potassium metabisulfite (0.09 g kg^−1^) was added to the samples to give a final total SO_2_ concentration of 50 mg L^−1^, and then musts were inoculated with 0.2 g kg^−1^ of commercial *Saccharomyces cerevisiae* r.f. *bayanus* (Enartis, Trecate, Italy). The must was fermented at a controlled temperature of 25 °C. The alcoholic fermentation finished when reducing sugars were below 2.5 g L^−1^ (two weeks after yeast inoculation). Then, the wine was separated from the seeds and skins by pressing, and the wine analysis was performed. 

Color intensity (CI), hue and total polyphenol index (TPI) were analyzed according to official methods [41]. Total phenols, antioxidant capacity, the bulk levels of UV-absorbing compounds (UVAC), individual phenolic compounds, and VOCs, were analyzed as in berry skins in previous studies [9,14]. Total phenols were determined using the Folin–Ciocalteu reagent and expressed as the gallic acid equivalents (GAE). The antioxidant capacity was measured by generating the radical cation 2,2′-azinobis(3-ethylbenzothiazoline-6-sulfonic acid) (ABTS•+), and expressed in terms of Trolox equivalents (TE). The bulk level of UV-absorbing compounds (UVAC) was measured as the area under the absorbance curve in two wavelength intervals, 280–315 and 280–400 nm, corresponding to UV-B and the sum of UV-B and UV-A (AUC_280–315_ and AUC_280–400_, respectively), using a Perkin Elmer λ35 spectrophotometer (Perkin Elmer, Wilton, CT, USA). Individual phenolic compounds were analyzed by UPLC/LC–MS (Waters Acquity UPLC system, Waters Corporation, Milford, MA, USA). Solvents were: A, water/formic acid (0.1%), and B, acetonitrile with 0.1% formic acid. The gradient program employed was: 0–7 min, 99.5—80% A; 7–9 min, 80—50% A; 9–11.7 min, 50—0% A; 11.7–15 min, 0—99.5% A. The UPLC system was coupled to a micrOTOF-QII-ESI-MS/MS high-resolution mass spectrometer (Bruker Daltonics, Bremen, Germany) controlled by the Bruker Daltonics Data Analysis software. The electrospray (ESI) source was operated in positive or negative mode, in the range of *m*/*z* 120 and 1505. The optimized conditions of the ESI source were as follows: capillary potential 4 kV, ESI source temperature 180 °C, desolvation temperature 200 °C, gas flow 9 L min^−1^; nebulizer gas 3.5 bar and 25 °C. LC–MS and MS/MS were performed operating in continuum mode. The spectra were acquired at two scans per second. The fragmentor voltage for MS/MS acquisition mode was 35 eV. The identity assignation of compounds was carried out by combining different information: retention time, UV-Vis data, MS spectra, MS/MS fragmentation patterns of peaks of available pure compounds, and literature data [42]. For quantification, DAD chromatograms were extracted at 520 nm for anthocyanins and 324 nm for the other compounds; the calibration curves of the respective standards were used. In absence of commercial standards, compounds with the same chromophore were used: stilbenes using *t*-resveratrol; flavanols using catechin, epigallocatechin, and procyanidin B1; flavonols using isorhamnetin-3-*O*-glucoside, kaempferol-3-*O*-glucoside, myricetin, quercetin, quercetin-3-*O*-galactoside, quercetin-3-*O*-glucoside, quercetin-3-*O*-glucuronide, and syringetin-3-*O*-glucoside; hydroxybenzoic acids using gallic acid; hydroxycinnamic acid derivatives using caffeic and *p*-coumaric acids, and anthocyanins using malvidin-3-*O*-glucoside (Sigma-Aldrich, St. Louis, MO, USA; Fluka, Buchs, Germany; Extrasynthese, Genay, France). The total contents of each phenolic group were obtained as the sum of their respective individual compounds. For VOC extraction, 1 mL of wine per sample was transferred to a 10 mL headspace screw cap vial and subjected to headspace solid-phase microextraction (HS-SPME). A 65 μM PDMS/DVB fiber (Supelco, Bellefonte, PA, USA) was used for this analysis. Pre-incubation and extraction were carried out at 50 °C for 10 and 20 min, respectively, followed by desorption for 1 min at 250 °C in the splitless mode. The VOCs trapped on the fiber were analyzed by GC–MS using an autosampler COMBI PAL CTC Analytics (Zwingen, Switzerland), a 6890 N GC Agilent Technologies (Santa Clara, CA, USA), and a 5975B Inert XL MSD Agilent equipped with an Agilent J&W Scientific DB-5 fused silica capillary column (5%-phenyl-95%-dimethylpolysiloxane as stationary phase, 60 m length, 0.25 mm i.d., and 1 μm thickness film). Oven temperature conditions were 40 °C for 2 min, 5 °C min^−1^ ramp up to 250 °C, followed by isothermal hold at 250 °C for 5 min. Helium was used as the carrier gas at 1.4 mL min^−1^ constant flow. *m*/*z* detection was obtained by an Agilent mass spectrometer operating in the EI mode (ionization energy of 70 eV; source temperature 230 °C), for which data were acquired in the scanning mode (mass range *m/z* 35–220). Chromatograms and spectra were recorded and processed using Enhanced ChemStation software for GC–MS (Agilent). Compound identification was based on the comparison between the MS for each putative compound with those of the NIST 2005 Mass Spectral library, as well as the match to a GC retention time and Mass Spectra custom library generated using commercially available compounds.

### 4.3. Statistical Analysis

Using a one-way analysis of variance (ANOVA), and once it was proven that the data met the assumptions of normality (Shapiro–Wilk test) and homoscedasticity (Levene’s test), we tested the global effect of the radiation regime to which grapes were exposed on the variables measured in the resulting wines. In the case of significant differences, means were then compared by Tukey test. The wine samples were ordinated through a principal components analysis (PCA), using the contents of individual phenolic compounds and VOCs. The PCA was performed using the data matrices of each biological replicate. All the statistical procedures were performed with SPSS 24.0 for Windows (SPSS Inc., Chicago, IL, USA).

## 5. Conclusions

To our knowledge, this is the first study demonstrating the significant influence of the different UV wavelengths and levels received by grapes on the phenolic and VOC composition of the resulting wines, despite the changes taking place during winemaking. In particular, flavonols were higher in wines whose grapes had been exposed to a combination of UV-A and UV-B radiation, while esters responded to UV-A alone. Overall, our results open up new possibilities for the artificial manipulation of UV radiation in grapevine cultivation, given the contribution of these compounds to wine quality, and the healthy properties of flavonols. Nevertheless, several years of experimentation would be needed to unequivocally confirm our results, thus avoiding the possible influence of the specific environmental conditions of the year when the experiment was performed. Future research should include experiments using different UV doses as well as high UV peak irradiances, to establish the treatments that most efficiently change grape and wine composition. In addition, other interacting factors could contribute to the further improvement of grape and wine quality [11,13,43,44].

## Figures and Tables

**Table 1:** Global variables (color intensity, hue, total polyphenol index, total phenols, antioxidant capacity, and bulk levels of UV-absorbing compounds (UVAC)) and individual phenolic compounds in wines made from grapes exposed to five different radiation regimes: P (only PAR), PA (PAR + UV-A), PB (PAR + UV-B), PAB (PAR + UV-A + UV-B), and PAB↑ (PAR + UV-A + enhanced UV-B). For each variable, the statistical significance of a one-way ANOVA test using the radiation regime as the main factor is shown, and different letters indicate significant differences between radiation regimes (Tukey test). Means ± SE are shown. *** *p* < 0.001; ** *p* < 0.01; * *p* < 0.05; ns, not significant. GAE, gallic acid equivalent. TE, Trolox equivalent. AUC_280–315_ and AUC_280–400_, area under the absorbance curve in the intervals 280–315 and 280–400 nm, respectively.

	P	PA	PB	PAB	PAB↑	Statistical Significance
Color intensity (CI)	14 ± 1	13 ± 0	14 ± 1	14 ± 0	13 ± 0	ns
Hue	0.76 ± 0.06	0.76 ± 0.07	0.73 ± 0.02	0.76 ± 0.05	0.72 ± 0.03	ns
Antioxidant capacity (mM TE)	24 ± 1 b	19 ± 0 a	23 ± 0 b	23 ± 1 b	22 ± 1 ab	**
Total polyphenol index (TPI)	60 ± 4 b	46 ± 2 a	55 ± 3 ab	54 ± 0 ab	50 ± 3 ab	*
Total phenols (GAE, g L^−1^)	2.6 ± 0.1	2.2 ± 0.1	2.7 ± 0.2	2.6 ± 0.1	2.5 ± 0.1	ns
UVAC (AUC_280–315_)	1523 ± 106	1231 ± 34	1425 ± 70	1394 ± 5	1281 ± 80	ns
UVAC (AUC_280–400_)	2616 ± 154	2336 ± 69	2626 ± 150	2655 ± 16	2390 ± 155	ns
*Stilbenes* (mg L^−1^)						
Resveratrol	0.09 ± 0.02	0.08 ± 0.01	0.12 ± 0.01	0.08 ± 0.01	0.12 ± 0.00	ns
Resveratrol-3-*O*-glucoside	1.7 ± 0.2	1.2 ± 0.1	2.1 ± 0.2	1.5 ± 0.1	1.7 ± 0.2	ns
*Flavanols* (mg L^−1^)						
Catechin	15 ± 2 b	8 ± 0 a	12 ± 1 ab	11 ± 1 ab	11 ± 1 ab	*
Epicatechin	6.7 ± 0.6	3.6 ± 0.2	5.3 ± 0.1	5.6 ± 0.9	5.0 ± 0.4	ns
Gallocatechin	0.90 ± 0.15	1.3 ± 0.2	0.99 ± 0.12	1.2 ± 0.2	1.3 ± 0.2	ns
Epigallocatechin	6.7 ± 1.8	10 ± 1	13 ± 0	13 ± 1	14 ± 2	ns
Catechin gallate	0.13 ± 0.03 ab	0.08 ± 0.01 ab	0.18 ± 0.03 b	0.05 ± 0.02 a	0.11 ± 0.02 ab	*
Procyanidin B1	8.0 ± 0.5	5.6 ± 0.8	8.7 ± 0.9	7.5 ± 0.4	8.2 ± 0.7	ns
Procyanidin B2	3.0 ± 0.3 b	1.4 ± 0.1 a	2.5 ± 0.0 ab	2.3 ± 0.3 ab	2.2 ± 0.3 ab	**
*Flavonols (mg L^−1^)*						
Kaempferol	0.42 ± 0.03 a	0.77 ± 0.13 a	1.90 ± 0.11 b	0.72 ± 0.18 a	0.68 ± 0.01 a	***
Kaempferol-3-*O*-glucoside	0.02 ± 0.00 a	0.06 ± 0.00 abc	0.04 ± 0.01 ab	0.14 ± 0.03 c	0.11 ± 0.04 bc	**
Kaempferol-3-*O*-glucuronide	0.02 ± 0.00 a	0.01 ± 0.00 a	0.02 ± 0.00 a	0.03 ± 0.00 b	0.04 ± 0.01 b	***
Myricetin	3.3 ± 0.2	3.8 ± 0.1	5.4 ± 0.7	6.0 ± 0.7	3.4 ± 1.0	ns
Myricetin-3-*O*-glucoside	3.0 ± 0.4	3.9 ± 0.1	4.0 ± 0.4	4.4 ± 0.4	4.6 ± 0.4	ns
Myricetin-3-*O*-glucuronide	1.4 ± 0.1 a	1.4 ± 0.0 a	1.5 ± 0.1 ab	1.8 ± 0.1 b	1.9 ± 0.2 b	*
Laricitrin	0.57 ± 0.06 ab	0.42 ± 0.05 a	0.58 ± 0.02 ab	0.66 ± 0.05 b	0.53 ± 0.04 ab	*
Laricitrin-3-*O*-glucoside	0.76 ± 0.13	0.63 ± 0.02	0.91 ± 0.15	0.84 ± 0.03	1.20 ± 0.12	ns
Quercetin	4.7 ± 1.6	1.4 ± 0.2	2.1 ± 0.3	4.2 ± 0.8	2.5 ± 0.5	*
Quercetin-3-*O*-galactoside	5.3 ± 0.1 a	8.6 ± 0.5 ab	8.6 ± 0.8 ab	13 ± 2 b	14 ± 1 b	**
Quercetin-3-*O*-glucoside	5.6 ± 0.4 a	3.5 ± 0.6 a	4.9 ± 0.7 a	13 ± 2 b	12 ± 1 b	***
Quercetin-3-*O*-glucuronide	3.0 ± 0.2 a	1.9 ± 0.2 a	2.7 ± 0.3 a	4.5 ± 0.3 b	5.0 ± 0.8 b	***
Isorhamnetin-3-*O*-glucoside	0.08 ± 0.01	0.09 ± 0.02	0.09 ± 0.01	0.11 ± 0.01	0.10 ± 0.01	ns
Isorhamnetin-3-*O*-glucuronide	0.87 ± 0.16 a	0.86 ± 0.06 a	1.12 ± 0.10 ab	1.71 ± 0.31 b	1.50 ± 0.22 ab	*
Syringetin	0.31 ± 0.06	-	-	0.16 ± 0.03	0.17 ± 0.01	ns
Syringetin-3-*O*-glucoside	2.2 ± 0.2	1.6 ± 0.1	2.0 ± 0.1	2.5 ± 0.3	2.3 ± 0.2	ns
*Hydroxybenzoic acids* (mg L^−1^)						
Protocatechuic acid	0.63 ± 0.06	0.33 ± 0.02	0.59 ± 0.03	0.73 ± 0.14	0.63 ± 0.03	ns
Gallic acid	10 ± 1	8 ± 0	10 ± 1	12 ± 1	11 ± 0	ns
*Hydroxycinnamic acid derivatives* (mg L^−1^)						
Caffeoyl tartaric acid	27 ± 5	31 ± 3	35 ± 4	35 ± 1	36 ± 2	ns
Coumaroyl tartaric acid	15 ± 4	17 ± 2	11 ± 0	12 ± 1	16 ± 2	ns
Caffeic acid ethyl ester	1.1 ± 0.2 ab	0.21 ± 0.00 a	0.61 ± 0.07 ab	0.80 ± 0.10 ab	1.5 ± 0.4 b	*
*Anthocyanins* (mg L^−1^)						
Cyanidin-3-*O*-glucoside	2.2 ± 0.2 b	0.82 ± 0.01 a	2.2 ± 0.3 b	1.8 ± 0.2 ab	2.1 ± 0.2 b	*
Delphinidin-3-*O*-glucoside	26 ± 1 ab	18 ± 5 a	60 ± 9 bc	48 ± 7 abc	73 ± 10 c	**
Malvidin-3-*O*-glucoside	156 ± 5	262 ± 26	230 ± 18	210 ± 33	295 ± 44	ns
Peonidin-3-*O*-glucoside	14 ± 2 b	3.4 ± 0.9 a	14 ± 2 b	12 ± 1 b	15 ± 0 b	**
Petunidin-3-*O*-glucoside	41 ± 5	60 ± 11	66 ± 4	66 ± 7	78 ± 11	ns
Cyanidin-3-*O*-(6′-acetyl)glucoside	1.7 ± 0.3	1.0 ± 0.2	1.8 ± 0.1	1.8 ± 0.0	2.0 ± 0.4	ns
Delphinidin-3-*O*-(6′-acetyl)glucoside	0.91 ± 0.18	1.5 ± 0.2	1.5 ± 0.1	1.4 ± 0.1	1.6 ± 0.1	ns
Malvidin-3-*O*-(6′-acetyl)glucoside	20 ± 2	37 ± 4	33 ± 4	28 ± 5	30 ± 3	ns
Peonidin-3-*O*-(6′-acetyl)glucoside	1.3 ± 0.1	0.94 ± 0.23	1.4 ± 0.2	1.2 ± 0.0	1.3 ± 0.1	ns
Petunidin-3-*O*-(6′-acetyl)glucoside	2.3 ± 0.7	2.9 ± 0.5	4.2 ± 0.4	3.7 ± 0.2	4.5 ± 0.6	ns
Cyanidin-3-*O*-(6′-p-coumaroyl)glucoside	4.3 ± 0.4	2.9 ± 0.7	5.5 ± 0.8	4.6 ± 0.2	4.5 ± 0.7	ns
Delphinidin-3-*O*-(6′-p-coumaroyl)glucoside	6.7 ± 1.2	14 ± 2	15 ± 2	14 ± 2	4.5 ± 0.3	ns
Malvidin-3-*O*-(6′-p-coumaroyl)glucoside	19 ± 3	34 ± 3	46 ± 2	29 ± 7	27 ± 3	ns
Peonidin-3-*O*-(6′-p-coumaroyl)glucoside	1.4 ± 0.2	1.0 ± 0.3	1.9 ± 0.3	1.4 ± 0.1	1.3 ± 0.1	ns
Petunidin-3-*O*-(6′-p-coumaroyl)glucoside	1.7 ± 0.3 a	3.6 ± 0.3 ab	4.1 ± 0.6 b	3.3 ± 0.5 ab	3.3 ± 0.8 ab	*

**Table 2:** Relative abundance (percentages) of individual volatile organic compounds (VOCs) in wines made from grapes exposed to five different radiation regimes: P (only PAR), PA (PAR + UV-A), PB (PAR + UV-B), PAB (PAR + UV-A + UV-B), and PAB↑ (PAR + UV-A + enhanced UV-B). For each compound, the statistical significance of a one-way ANOVA test using the radiation regime as the main factor is shown, and different letters indicate significant differences between radiation regimes (Tukey test). Means ± SE are shown. *** *p* < 0.001; ** *p* < 0.01; * *p* < 0.05; ns, not significant.

	P	PA	PB	PAB	PAB↑	Statistical Significance
*Alcohols*						
2-Methyl-1-butanol	19 ± 2 a	25 ± 3 ab	19 ± 2 a	20 ± 2 a	34 ± 2 b	**
3-Methyl butanol	25 ± 1 b	14 ± 2 a	24 ± 1 b	21 ± 1 b	14 ± 0 a	***
1-Hexanol	1.0 ± 0.1	1.0 ± 0.1	1.1 ± 0.1	1.2 ± 0.1	1.0 ± 0.1	ns
Phenyl ethyl alcohol	21 ± 2	18 ± 2	22 ± 2	25 ± 2	22 ± 1	ns
*Hydrocarbons*						
Tridecane	0.02 ± 0.00	0.02 ± 0.00	0.02 ± 0.00	0.02 ± 0.00	0.02 ± 0.00	ns
Tetradecane	0.03 ± 0.00 a	0.05 ± 0.00 ab	0.04 ± 0.00 a	0.05 ± 0.00 ab	0.06 ± 0.01 b	*
*Esters*						
Acetic acid, 2-phenylethyl ester	0.09 ± 0.01	0.10 ± 0.01	0.10 ± 0.00	0.12 ± 0.00	0.11 ± 0.01	ns
Diethyl succinate	0.70 ± 0.13	0.72 ± 0.05	0.85 ± 0.07	0.79 ± 0.14	0.55 ± 0.04	ns
Ethyl acetate	6.5 ± 1.0	6.4 ± 0.7	6.5 ± 0.4	6.0 ± 0.9	4.3 ± 0.5	ns
Ethyl butanoate	0.29 ± 0.04	0.43 ± 0.05	0.43 ± 0.07	0.35 ± 0.03	0.34 ± 0.04	ns
Ethyl decanoate	0.97 ± 0.13 a	2.1 ± 0.0 c	1.2 ± 0.2 ab	1.1 ± 0.1 ab	1.7 ± 0.3 bc	**
Ethyl dodecanoate	0.52 ± 0.06 c	0.30 ± 0.02 ab	0.43 ± 0.02 bc	0.26 ± 0.01 a	0.20 ± 0.03 a	***
Ethyl heptanoate	0.11 ± 0.01	0.06 ± 0.01	0.09 ± 0.01	0.09 ± 0.01	0.06 ± 0.01	*
Ethyl hexanoate	7.1 ± 0.6	8.2 ± 0.5	6.4 ± 0.5	7.2 ± 0.5	6.2 ± 0.9	ns
Ethyl hexyl salicylate	0.10 ± 0.01	0.06 ± 0.00	0.10 ± 0.03	0.07 ± 0.01	0.07 ± 0.01	ns
Ethyl nonanoate	1.2 ± 0.1 b	1.3 ± 0.1 b	1.1 ± 0.1 ab	1.3 ± 0.2 b	0.63 ± 0.02 a	*
Ethyl octanoate	8.4 ± 1.4 a	16 ± 1 b	8.9 ± 1.0 a	10 ± 1 ab	13 ± 2 ab	*
Hexadecanoic acid, ethyl ester	4.7 ± 0.2	3.0 ± 0.1	4.8 ± 1.0	2.7 ± 0.6	2.1 ± 1.8	ns
2-Methylbutyl acetate	0.23 ± 0.05	0.20 ± 0.01	0.20 ± 0.01	0.21 ± 0.01	0.16 ± 0.02	ns
3-Methylbutyl acetate	0.64 ± 0.10	0.88 ± 0.02	0.86 ± 0.08	0.91 ± 0.06	0.68 ± 0.11	ns
Methyl hexanoate	0.03 ± 0.00	0.03 ± 0.00	0.03 ± 0.00	0.04 ± 0.00	0.02 ± 0.01	ns
Methyl octanoate	0.08 ± 0.01 a	0.13 ± 0.02 ab	0.09 ± 0.01 a	0.11 ± 0.02 ab	0.18 ± 0.01 b	*
Octanoic acid, 3-methylbutyl ester	0.04 ± 0.00	0.07 ± 0.01	0.04 ± 0.00	0.04 ± 0.00	0.05 ± 0.01	*
Tetradecanoic acid ethyl ester	0.50 ± 0.08 c	0.12 ± 0.02 ab	0.32 ± 0.05 bc	0.10 ± 0.02 ab	0.05 ± 0.00 a	***
*Fatty acids*						
Ethanoic acid	0.95 ± 0.25	0.81 ± 0.07	0.71 ± 0.21	0.83 ± 0.30	0.50 ± 0.05	ns
Hexanoic acid	0.46 ± 0.04 a	0.73 ± 0.05 b	0.42 ± 0.01 a	0.59 ± 0.05 ab	0.60 ± 0.08 ab	*
Nonanoic acid	0.07 ± 0.00 a	0.21 ± 0.01 b	0.09 ± 0.01 a	0.12 ± 0.01 ab	0.31 ± 0.05 c	***
Octanoic acid	0.22 ± 0.03 a	0.70 ± 0.02 c	0.31 ± 0.03 ab	0.45 ± 0.05 abc	0.57 ± 0.11 bc	**
*Other compounds*						
Hydroxybutyric acid lactone	0.03 ± 0.01	0.02 ± 0.00	0.03 ± 0.00	0.03 ± 0.00	0.02 ± 0.00	ns
n-Nonaldehyde	0.08 ± 0.00 bc	0.06 ± 0.00 ab	0.07 ± 0.00 b	0.09 ± 0.01 c	0.05 ± 0.00 a	*

**Table 3:** A comparison of the effects of UV radiation on 51 variables measured in both grapes [9] and the resulting wines (this study). Grapes were exposed to five different radiation regimes: P (only PAR), PA (PAR + UV-A), PB (PAR + UV-B), PAB (PAR + UV-A + UV-B), and PAB↑ (PAR + UV-A + enhanced UV-B). Ns, no significant effect of radiation regime in one-way ANOVA. Nd, significant effect of radiation regime in one-way ANOVA, but no significant differences between treatments in a post hoc Tukey test. In bold type, variables showing similar significant responses in both grapes and wines.

Variable	Effect on Grapes	Effect on Wines
Stilbenes	PA, PB, PAB > P	PB, PAB↑ > PA
Flavanols	PAB > P, PA	PB > PA
**Flavonols**	**PAB, PAB** **↑** **> P, PA, PB**	**PAB, PAB** **↑** **> P, PA, PB**
Hydroxycinnamic acids	PAB > PA	PA, PAB, PAB↑ > P
Anthocyanins	Ns	Ns
Alcohols	Ns	P > PA
Hydrocarbons	PAB↑ > P, PA, PB, PAB	Ns
Aldehydes	Ns	P > PA, PAB↑
Fatty acids	PB, PAB↑ > P, PA, PAB	Ns
Total phenols	Ns	Ns
Antioxidant capacity	Ns	P, PB, PAB > PA
UV-absorbing compounds	Ns	Ns
Resveratrol-3-*O*-glucoside	PA, PAB > P, PAB↑	Ns
Catechin	Ns	P > PA
Epicatechin	Ns	Ns
Catechin gallate	Ns	PB > PAB
Procyanidin B1	Nd	Ns
Kaempferol	Ns	PB > remaining regimes
**Kaempferol-3-*O*-glucoside**	**PAB > PA, PB**	**PAB > PB, P**
Myricetin	Ns	Ns
Myricetin-3-*O*-glucoside	Ns	Ns
Myricetin-3-*O*-glucuronide	Ns	PAB, PAB↑ > P, PA
**Quercetin-3-*O*-galactoside**	**PAB > P, PA, PB**	**PAB, PAB** **↑ > P**
**Quercetin-3-*O*-glucoside**	**PAB > PA**	**PAB, PAB** **↑** **> P, PA, PB**
**Quercetin-3-*O*-glucuronide**	**PAB > P, PA, PB**	**PAB, PAB** **↑** **> P, PA, PB**
Isorhamnetin-3-*O*-glucoside	PAB > P, PA	Ns
Isorhamnetin-3-*O*-glucur.	Nd	PAB > P, PA
Syringetin-3-*O*-glucoside	PAB > P	Ns
Caffeoyl tartaric acid	Nd	Ns
Coumaroyl tartaric acid	Nd	Ns
Cyanidin-3-*O*-glucoside	Ns	P, PB, PAB↑ > PA
Delphinidin-3-*O*-glucoside	Ns	PAB↑ > P, PA
Peonidin-3-*O*-glucoside	Ns	P, PB, PAB, PAB↑ > PA
Petunidin-3-*O*-6′-coum-glucos	Ns	PB > P
Remaining 11 anthocyanins	Ns	Ns
1-Hexanol	Ns	Ns
Tridecane	Ns	Ns
Hexanoic acid	Ns	PA > P, PB
**Nonanoic acid**	**PB, PAB** **↑** **> P, PA, PAB**	**PAB** **↑ > remaining regimes**
Octanoic acid	PB, PAB↑ > P, PA, PAB	PA > P, PB
n-Nonaldehyde	Ns	PAB, P, PB > PAB↑

## Data Availability

Data supporting reported results is contained within the article.
